# Case Report: Diagnosis, treatment and follow-up of venous thrombosis in adolescent diabetic patients with SARS-CoV-2

**DOI:** 10.3389/fped.2025.1562590

**Published:** 2025-08-11

**Authors:** Fanshu Ma, Qiang Zhang, Li Pei, JinFeng Shuai, Jinying Li

**Affiliations:** ^1^ Department of Endocrinology, Genetics and Metabolism, Children’s Hospital of Hebei Provience, Hebei Medical University, Shijiazhuang, China; ^2^ Department of Respiratory, Children’s Hospital of Hebei Provience, Hebei Medical University, Shijiazhuang, China; ^3^ Department of Pediatric Medicine, Shanghai University of Medicine & Health Sciences Affiliated Zhoupu Hospital, Shanghai, China

**Keywords:** diabetes, children, vein thrombosis, SARS-CoV-2, follow-up

## Abstract

The highest risk of diabetes mellitus (DM)-related complications, collectively known as venous thromboembolism (VTE), is observed in the age group of 20–39 years. However, this observation sharply contrasts with the increased incidence of superficial venous thrombosis observed among adolescent patients during the coronavirus disease 2019 (COVID-19) outbreak in Hebei Province in December 2022. Moreover, it contradicts the absence of venous thrombosis observed in pediatric diabetic patients who were treated prior to the COVID-19 outbreak. This study collected and analyzed clinical data related to diabetic venous thrombosis in children, with the aim of provide evidence for the prevention and treatment of this complication.

## 1 Introduction

Population studies predict that more than 600 million individuals will be diagnosed with diabetes by 2042 (1). Diabetes-related complications are responsible for 6% of global mortality, accounting for over 3 million deaths per year. Diabetic patients exhibit a mortality rate from cardiovascular diseases that is two to four times higher than that of the general population (2). Diabetes has been identified as a risk factor for VTE. The intricate metabolic disturbances associated with diabetes often lead to long-term complications affecting both macrovascular and microvascular systems. A retrospective cohort study (3) demonstrated that individuals with type 1 diabetes mellitus (T1DM) have a 5.33 times greater risk of developing VTE compared to those without T1DM, thus confirming T1DM as an independent risk factor for VTE. Despite T1DM comprising only 5%–10% of all diabetes cases, its onset typically occurs earlier than type 2 diabetes mellitus (T2DM), suggesting that it may precipitate related complications at an earlier stage and have a more detrimental impact on the quality of life of patients.

COVID-19, caused by the severe acute respiratory syndrome coronavirus 2 (SARS-CoV-2), can lead to excessive inflammatory responses, hypoxia, endothelial damage, and both localized and disseminated intravascular coagulation in severe cases. Co-infection with COVID-19 exacerbates pre-existing endothelial dysfunction and microvascular damage in diabetic patients, thereby intensifying the severity of the infection. Furthermore, in patients with diabetes, infection with SARS-CoV-2 induces a more pronounced inflammatory response due to the excessive production of interleukins—specifically interleukin-6 (IL-6), interleukin-8 (IL-8), and tumor necrosis factor-α (TNF-α). This heightened inflammatory state leads to lymphocyte apoptosis, suppression of innate immunity, and further activation of the coagulation cascade, ultimately resulting in an increased risk of cardiovascular mortality in diabetic patients.

According to the study by Tang et al. (4), significant differences in coagulation indicators have been observed between deceased and surviving patients with COVID-19. Specifically, D-dimer levels were elevated approximately 3.5-fold, fibrin degradation products increased by approximately 1.9-fold, and prothrombin time (PT) was significantly prolonged (*P* < 0.001). Consequently, the imbalance between coagulation factors and fibrinolysis may contribute to an increased risk of thrombotic events and subsequent mortality (5). This article presents a case series of six adolescent patients with diabetes who developed venous thromboembolism following a COVID-19 infection. The study analyzes their clinical features, diagnostic procedures, and treatment processes, with the aim of identify early warning signs of venous thrombosis in diabetic children. It is hoped that this research will increase clinicians' awareness of thrombosis-related complications in pediatric diabetes patients.

## 2 Patients and methods

### 2.1 Ethics approval

The study was approved by the Institutional Ethics Committee of the Children's Hospital of Hebei Province. The patients' parents provided informed consent for participation in this study and the publication of the results.

### 2.2 Patients

**Figure 1:**
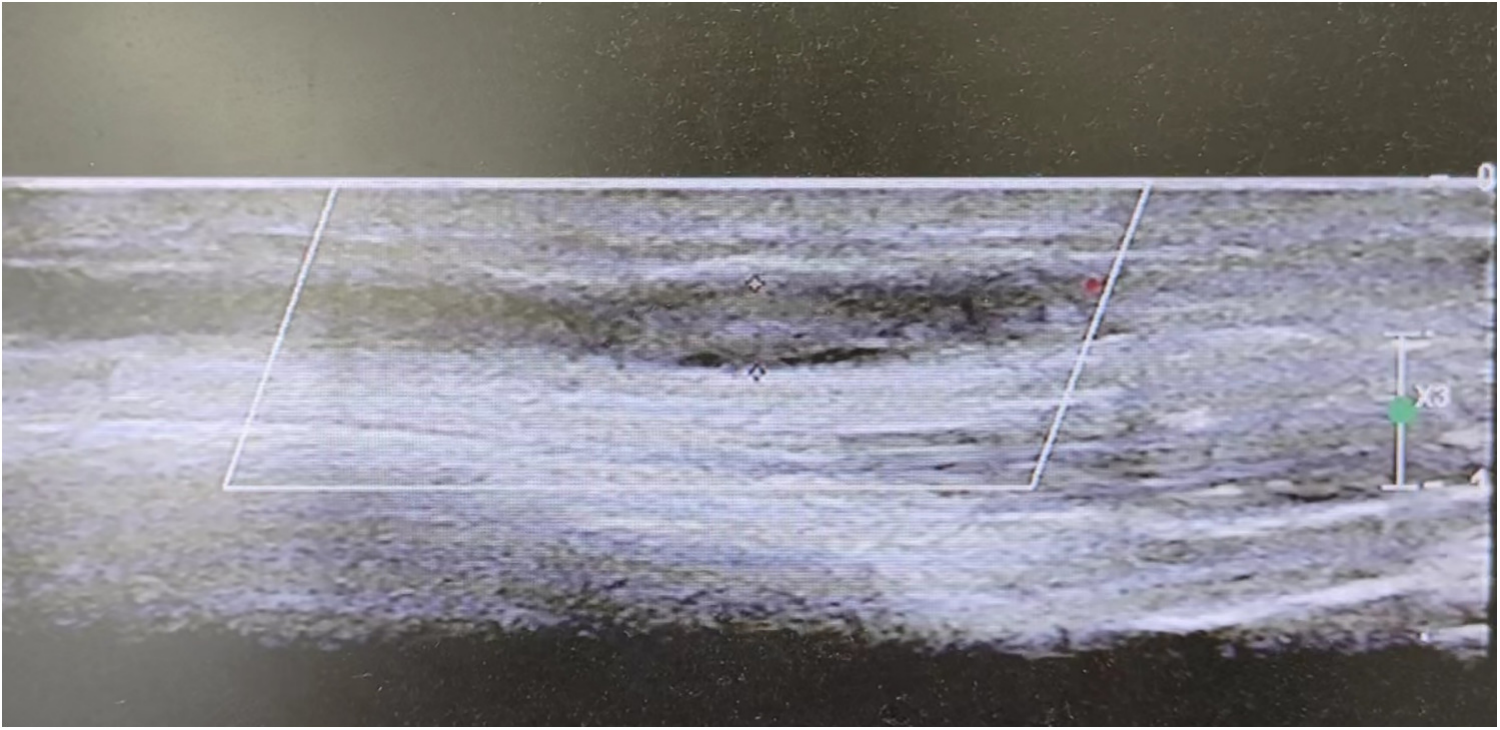
Ultrasound of superficial veins shows the formation of thrombus of patient 1.

Between December 2022 and February 2023, six diabetic patients were admitted to the Endocrinology and Genetic Metabolism Department at the Children's Hospital of Hebei Province. Five of these patients were admitted with diabetic ketoacidosis (DKA), and one patient presented with left lower limb pain. All patients had been diagnosed with T1DM. Some patients had contracted COVID-19 (Omicron variant) prior to admission, while others were infected during their hospital stay. Unfortunately, the vaccination status of these children against COVID-19 could not be ascertained due to data limitations. All patients exhibited symptoms of limb pain, and ultrasound examinations confirmed the presence of venous thrombosis (Figure 1).

Six children, aged between 10 and 16 years, were included, with one child having been diagnosed with diabetes and five presenting as new cases. Five of these children were admitted with DKA and two exhibited significantly elevated white blood cell counts; however, five had the C-reactive protein (CRP) levels that remained within normal limits. Admission blood glucose levels ranged from 12.8 mmol/L to 41 mmol/L (normal range: 3.9–6.1 mmol/L). Throughout hospitalization, coagulation function was not significantly altered in any of the six patients, and plasma D-dimer levels were within normal ranges. During hospitalization, all six children experienced limb pain, and ultrasound examination revealed the presence of superficial venous thrombosis. Patients with more severe symptoms were treated with low-molecular-weight heparin (LMWH), while those with milder symptoms received topical polysulfonated mucopolysaccharide cream. After a six-month follow-up, one patient experienced worsening symptoms, one fully recovered, and four showed improvement in symptoms (with reduced vein width). One year later, a questionnaire survey revealed that four patients had fully recovered, one developed collateral vessel formation with no blood flow observed within the original vessel, and one patient with worsening symptoms was re-evaluated at another hospital and gradually recovered after treatment. Two years later, a follow-up questionnaire survey indicated that five patients had fully recovered, and the patient with collateral vessel formation showed no significant changes (Table 1).

**Table 1:** Clinical characteristics, treatment and follow-up of six diabetic patients with venous thrombosis.

Clinical information	Patient 1	Patient 2	Patient 3	Patient 4	Patient 5	Patient 6	Reference range
Age (years old)	11	12	16	13	13	10	
Sex	Female	Male	Male	Male	Female	Male	
BMI (kg/m^2^)	16.4	14.6	13.3	14.9	15.4	18	
Date of illness	2022/12/1	2022/12/22	2022/12/20	2015/6/18	2022/12/7	2022/11/5	
Date of infection with COVID-19	2022/12/9	2022/12/12	–	2022/12/14	2022/12/21	2022/12/8	
Date of VTE	2022/12/26	2023/2/1	2023/2/7	2022/12/27	2023/1/1	2022/12/28	
Incipient symptom	Pain	Pain	Pain	Pain	Pain	Pain	
WBC (×10^9^/L)	23.7	6.8	5.9	31.3	5.5	11.8	4.00–10.00
HGB (g/L)	160	147	155	159	139	122	110–160
PLT (×10^9^/L)	444	293	462	384	253	231	100–300
NE (%)	86.4	53.9	53.4	74.2	41.7	77.8	50.0–70.0
LY (%)	9.4	38.3	40.1	20.4	48.6	12.4	20.0–40.0
CRP (mg/L)	<0.6	<0.5	0.35	1.64	1.4	13.02	0–10.0
Admission blood glucose level (mmol/L)	41	18.7	12.8	41	36	17.3	3.9–6.1
C-Peptide	0.13	0.35	<0.003	0.01	0.19	0.1	0.37–1.47
Insulin	16.28	82.57	11.17	11.4	44.94	10.1	17.8–173.0
GHB (%)	12.8	12.3	9.7	14.1	12.3	15.5	4.0–6.0
PT	10.9	10.4	9.5	10.8	10.0	10.6	9.8–12.1
INR	0.95	0.90	0.82	0.94	0.87	0.92	0.80–1.20
APTT	24.9	23.7	23.6	26.1	29.3	23.6	25.0–31.0
TT	17.6	20.4	18.1	15.8	15.8	16.3	14.0–21.0
FIB (g/L)	2.05	1.71	2.3	2.92	2.92	2.13	1.8–3.5
D-dimer (mg/L)	0.57	0.59	<0.19	0.29	0.2	0.72	0–0.55
DKA	yes	yes	no	yes	yes	yes	
pH	6.844	7.298	7.381	6.905	7.219	7.026	7.350–7.450
Indwelling needle position	Right elbow	Right elbow and right foot	–	Right wrist	Left hand	Right elbow	
DM family history	No	No	No	No	Yes	Yes	
Therapy	LMWH, Mucopolysaccharide Polysulfate cream	LMWH	LMWH	Mucopolysaccharide Polysulfate cream	Mucopolysaccharide Polysulfate cream	Observe	
Sonographic examination	Patient 1	Patient 2	Patient 3	Patient 4	Patient 5	Patient 6	
1-month follow-up	Improve	Improve	Improve	No change	Improve	Worsen	
3-month follow-up	Improve	Improve	Improve	Collateral formation	Improve	Worsen	
6-month follow-up	Disappear	Improve	Improve	Collateral formation	Improve	Worsen	
1-year follow-up	–	Disappear	Disappear	Collateral formation	Disappear	Improve	
2-year follow-up	–	–	–	Collateral formation	–	Disappear	

### 2.3 Hematological index measurement

Blood tests were performed according to standard methods. Upon admission, the following parameters were assessed: blood routine, C-reactive proteinblood (CRP), glucose levels, C-peptide, insulin, glycosylated hemoglobin (GHB), coagulation function, D-dimer levels, and pH values (Table 1).

## 3 Results

All six patients were diagnosed with TMD1, five of whom were admitted due to DKA. Upon admission, all patients exhibited elevated blood glucose levels, with two showing significantly elevated white blood cell counts and one presenting with elevated CRP. Coagulation function and D-dimer levels, however, did not exhibit any significant abnormalities. Following the contraction of COVID-19, all these patients experienced limb pain, and ultrasound examination revealed the presence of superficial venous thrombosis. After treatment and a 2-year follow-up, five patients fully recovered, while one developed collateral vessel formation.

## 4 Discussion

In the general population, some studies suggest that there is no significant association or only a slight positive correlation between DM and VTE (6). However, in patients with diabetes and concurrent COVID-19 infection, an increased incidence of VTE has been observed (7). This finding aligns with our observations of an elevated incidence of VTE in diabetic patients following COVID-19 infection. During the COVID-19 pandemic, six adolescent patients with diabetes and COVID-19 were documented to have developed superficial venous thrombosis, a phenomenon not observed in the 640 diabetic patients treated over the past five years, indicating a strong association with COVID-19 infection.

It has been established that COVID-19 is associated with heightened coagulation activity, frequently manifesting as a hypercoagulable state (8). The initial detection of clots was prompted by limb pain in affected children, with some cases severe as to disrupt sleep. Blood clots were identified through ultrasound examinations conducted after the onset of pain at the clot site. Notably, one child exhibited no limb pain during hospitalization; instead, routine ultrasound screening for vascular complications revealed thickening of the calf wall of the right great saphenous vein and an irregular echo. Subsequently, this child developed pain and venous thrombosis in the lower limb10 days later, underscoring the significance of vascular wall thickening and irregular echo detected by ultrasound as critical early warning signs of thrombosis. The prevalence of venous thrombosis is notably higher in diabetic patients concurrently infected with COVID-19, the need for routine ultrasound screenings to rule out thrombosis.

Hyperglycemia at hospital admission in adult patients with DM (regardless of diabetes subtype) following SARS-CoV-2 infection has been identified as a significant predictor of mortality and severe complications (9, 10). A statistical analysis (10) conducted by the Spanish Society of Internal Medicine involving 11,312 hospitalized COVID-19 patients (18.9% with diabetes) demonstrated that patients presenting with hyperglycemia at admission, regardless of prior diabetes diagnosis, had an increased risk of complications and death, with the risk rising with the severity of hyperglycemia [blood glucose >180 mg/dl (>10 mmol/L)]. In our study cohort, all adolescent patients with SARS-CoV-2 infection exhibited admission blood glucose levels exceeding 10 mmol/L, with three cases showing severe hyperglycemia >35 mmol/L. The mean glucose level in pediatric patients with venous thrombosis was 27.8 mmol/L, with no statistically significant difference compared to diabetic children with COVID-19 without thrombosis (26.1 mmol/L, *n* = 3; *P* = 0.067). Therefore, hyperglycemia should not be considered an independent risk factor for venous thrombosis. However, this result may reflect statistical limitations arising from the modest sample size in our study. According to Letko et al. (11), SARS-CoV-2 enters host cells via the angiotensin-converting enzyme 2 (ACE2) receptor, expressed in pancreatic β-cells. Following viral entry into the islets, β-cells are damaged, impairing insulin secretion and leading to insulin deficiency. This exacerbates the progression of diabetes and induces acute hyperglycemia, even in patients without pre-existing DM (12). Furthermore, acute hyperglycemia upregulates ACE2 expression in cells, potentially facilitating viral entry (9). Subsequent extensive viral invasion further destroys β-cells, elevating blood glucose levels and perpetuating a vicious cycle. In our study, all six patients were diagnosed with T1DM, which characterized by the destruction of pancreatic β-cells and absolute insulin deficiency. Consequently, patients with T1DM co-infected with COVID-19 are at a higher risk of developing severe complications.

Due to excessive inflammation, hypoxia, and both localized and disseminated intravascular coagulation, COVID-19 increases the risk of venous and arterial thromboembolism. The direct invasion of SARS-CoV-2 causes acute vascular endothelial damage, followed by viral replication within the cells, resulting in the release of mature viral particles into circulating immune cells (CD3, CD4, and CD8T cells) (13). This process impairs mitochondrial function and suppresses the T cell immune response to viral infection. The resulting cytokine storm exacerbates systemic inflammation. Elevated concentrations of inflammatory markers in the blood (such as C-reactive protein, procalcitonin, and ferritin), increased neutrophil-to-lymphocyte ratios, and higher levels of inflammatory cytokines and chemokines are strongly associated with the severity of COVID-19 (14–16). In patients with T1DM, significantly elevated levels of procoagulant mediators[von Willebrand factor (vWF), Factor V, VIII, X, and XI], indicate a hypercoagulable state, likely underpinned by autoimmune-mediated chronic inflammation (17). Research has demonstrated that, compared to COVID-19 patients without diabetes, those with diabetes exhibit a higher incidence of lymphopenia (44.5% vs. 32.6%) and elevated levels of inflammatory markers (C-reactive protein: 57.0% vs. 42.4%, procalcitonin: 33.3% vs. 20.3%) (18). Consequently, among COVID-19 patients, individuals with diabetes are more vulnerable to the detrimental effects of cytokine storms than those without diabetes.

Endothelial dysfunction associated with hypoxia intravascular coagulation during COVID-19 infection. ACE2 is highly expressed in endothelial cells (19). Consequently, the entry of SARS-CoV-2 into endothelial cells lead to their dysfunction or death, exposing the extracellular matrix to circulating blood and facilitating platelet adhesion (8). In our study, half of the patients exhibited elevated platelet count (normal range: from 100 × 10^9^/L to 300 × 10^9^/L), with the increase ranging from 1.3 to 1.5 times the upper limit of the normal range. Focusing on coagulation and platelet reactivity, diabetes predisposes individuals to VTE (20). Diabetes is characterized as a hypercoagulable state, where elevated plasma glucose levels influence the expression of platelet receptors and key enzymes during the megakaryocyte stage. Specifically, this involves a reduction in the expression of prostacyclin receptors (21), an upregulation of insulin-like growth factor 1 receptors (22), and an increase in the levels of the pro-oxidant enzyme nicotinamide adenine dinucleotide phosphate (NADPH) oxidase 1 (NOX1) (23). These changes collectively contribute to platelet hyperreactivity. Consequently, platelet hyperactivity is recognized as a critical driver of cardiovascular complications associated with diabetes (24, 25). Hyperglycemia results in elevated levels of advanced glycation end products (AGEs) in plasma and endothelial cells, which enhance the activity of endothelial receptors for advanced glycation end products (RAGE), leading to the production of reactive oxygen species (ROS) (26). The accumulation of ROS within the body or cells triggers cytotoxic responses and tissue damage, ultimately resulting in endothelial dysfunction and thrombosis. Research has demonstrated (27) that oxidative stress plays a significant role in the pathogenesis of various thrombotic conditions, including arterial thrombosis (e.g., atherosclerosis, ischemic stroke, myocardial infarction) and venous thrombotic disorders (e.g., deep vein thrombosis, pulmonary embolism). SARS-CoV-2 invades cells via the ACE2 receptor, resulting in mitochondrial dysfunction and abnormal accumulation of ROS. Simultaneously, the occupation of ACE2 by the virus prevents the hydrolysis of angiotensin II (Ang II), while the excessive production of Ang II enhances the activation of NADPH oxidase, a key enzyme responsible for ROS generation (28). These two mechanisms mediate a robust oxidative stress response. Oxidative stress not only exacerbates the inflammatory response induced by SARS-CoV-2 but also promotes the formation of neutrophil extracellular traps (NETs), which are closely associated with the development of immuno-thrombosis in severe COVID-19 (29). The interplay between inflammation and thrombosis further triggers oxidative stress, and these three factors collectively contribute to the cytokine storm, coagulation dysfunction, and tissue damage.

The chronic complications of diabetes mellitus include diabetic retinopathy, diabetic nephropathy, and diabetic neuropathy. Hyperglycemia induces dysregulation in metabolic pathways (polyol, AGEs, PKC, hexosamine), which directly damage neural structure and function. These pathways act synergistically, and vascular injury, coupled with ischemic hypoxia, further exacerbates neural damage. Persistent oxidative stress and inflammatory insults ultimately lead to peripheral neuropathy. An Italian research team reported two pediatric cases of abrupt-onset Pediatric Acute-onset Neuropsychiatric Syndrome (PANS), occurring 2 weeks post-SARS-CoV-2 infection, distinct from streptococcus-triggered PANDAS (as evidenced by negative anti-streptococcal antibodies). They postulated three potential mechanisms for SARS-CoV-2-induced PANS (direct viral neural invasion, vascular endothelial injury, autoimmune-mediated inflammatory responses), suggesting that COVID-19 may serve as an independent trigger (30). In our study, one child with pre-existing diabetes developed small fiber neuropathy (SFN) in the extremities following SARS-CoV-2 infection. However, attribution to either the virus or underlying diabetes could not be definitively established. Another child with pre-existing peripheral neuropathy showed no worsening of neurological deficits post-infection. The remaining children did not develop peripheral neuropathy. Furthermore, children with COVID-19 may develop complications, including multisystem inflammatory syndrome (MIS-C), cardiovascular injury, renal impairment, encephalitis, and seizures. The same research team documented cases of SARS-CoV-2-induced myositis in pediatric patients, characterized by symmetrical calf pain and gait abnormalities. A statistically significant correlation was observed between the urea/creatinine ratio (UCR) and creatine kinase (CK) normalization time, demonstrating immediate clinical applicability for monitoring disease progression (31).

D-dimer has been extensively investigated due to the procoagulant state associated with COVID-19 (32). This study demonstrated that D-dimer levels were moderately elevated in approximately half of the patients with venous thrombosis, while the remaining patients had levels within the normal range. Consequently, the utility of D-dimer as an early indicator for venous thrombosis is limited. However, elevated D-dimer levels should still be considered indicative of potential thrombus formation.

All diabetic patients with venous thrombosis in our study had T1DM. This finding aligns is consistent with prior research, which indicates that individuals with T1DM have a higher risk of VTE compared to those with T2DM (33). The underlying mechanism may involve acute β-cell dysfunction induced by SARS-CoV-2, which contributes to the development of insulin-deficient diabetes. Consequently, T1DM is considered an independent risk factor for venous thrombosis.

Given the evident pain symptoms in children following thrombosis, anticoagulation therapy may be essential for controlling and reducing pre-thrombotic events, as well as preventing pulmonary embolism. A review and comparison of coagulation test parameters and clinical characteristics among 449 severe COVID-19 patients, including both survivors and non-survivors, was conducted by Ning Tang et al. (34). These patients were stratified according to their D-dimer levels. Elevated D-dimer levels were associated with increased mortality among patients who did not receive heparin. Conversely, in patients who received heparin, D-dimer levels exceeding 3.0 µg/ml were linked to a 20% reduction in mortality. Therefore, heparin treatment in severe COVID-19 patients with coagulation dysfunction appears to be associated with a better prognosis. European authors recommend that, in patients with COVID-19 and D-dimer levels <0.5 mg/ml, prophylactic dose anticoagulation (enoxaparin 40 mg every 24 h) be administered; if D-dimer levels are between 0.5 and 3.0 µg/ml, enoxaparin 40 mg every 12 h be given; and if D-dimer levels exceed 3.0 mg/ml, enoxaparin 1 mg/kg every 12 h be used (35) Due to the anti-inflammatory properties of LMWH, three patients were administered LMWH therapy. The duration of LMWH treatment for pediatric thrombosis cases ranged from 7 to 14 days. After several months of follow-up, symptom improvement was observed in three patients treated with LMWH and two patients treated with mucopolysaccharide polysulfate cream exhibited symptom improvement. One patient with venous dilation presented with mild symptoms and was initially monitored clinically. During subsequent follow-up, this patient developed an extensive thrombus with minimal blood flow within the vessel. Consequently, in non-critically ill patients hospitalized due to COVID-19, therapeutic doses of heparin may reduce the need for organ support and the risk of progression to intubation or death, irrespective of D-dimer levels (36). Recent studies indicate that patients with diabetes have a higher predisposition to thrombosis,a common complication associated with COVID-19. Therefore, in the absence of contraindications, all diabetic patients hospitalized due to COVID-19 should be considered for prophylactic treatment against VTE (37).

In summary, DM is one of the most prevalent comorbidities in individuals with COVID-19 and a significant risk factor for adverse outcomes. Further research is essential to elucidate the pathophysiological mechanisms underlying the poor prognosis of diabetic patients with COVID-19, including the endothelial and microvascular dysfunction characteristic of diabetes. While the precise pathophysiological and molecular interactions between these two conditions remain incompletely understood, current evidence indicates that diabetes significantly affects the course of COVID-19 infection and exacerbates its clinical severity. Given the rising incidence VTE in diabetic patients with COVID-19, we recommend routine vascular ultrasound screening and early implementation of VTE prophylaxis are recommended for this patient population.

## Data Availability

The original contributions presented in the study are included in the article/Supplementary Material, further inquiries can be directed to the corresponding author.
